# Compressive Sensing-Based SAR Image Reconstruction from Sparse Radar Sensor Data Acquisition in Automotive FMCW Radar System

**DOI:** 10.3390/s21217283

**Published:** 2021-11-01

**Authors:** Seongwook Lee, Yunho Jung, Myeongjin Lee, Wookyung Lee

**Affiliations:** School of Electronics and Information Engineering, College of Engineering, Korea Aerospace University, Deogyang-gu, Goyang-si 10540, Gyeonggi-do, Korea; yjung@kau.ac.kr (Y.J.); artistic@kau.ac.kr (M.L.); wklee@kau.ac.kr (W.L.)

**Keywords:** compressive sensing, frequency-modulated continuous wave, range migration algorithm, synthetic aperture radar

## Abstract

In this paper, we propose a method for reconstructing synthetic aperture radar (SAR) images by applying a compressive sensing (CS) technique to sparsely acquired radar sensor data. In general, SAR image reconstruction algorithms require radar sensor data acquired at regular spatial intervals. However, when the speed of the radar-equipped platform is not constant, it is difficult to consistently perform regular data acquisitions. Therefore, we used the CS-based signal recovery method to efficiently reconstruct SAR images even when regular data acquisition was not performed. In the proposed method, we used the l1-norm minimization to overcome the non-uniform data acquisition problem, which replaced the Fourier transform and inverse Fourier transform in the conventional SAR image reconstruction method. In addition, to reduce the phase distortion of the recovered signal, the proposed method was applied to each of the in-phase and quadrature components of the acquired radar sensor data. To evaluate the performance of the proposed method, we conducted experiments using an automotive frequency-modulated continuous wave radar sensor. Then, the quality of the SAR image reconstructed with data acquired at regular intervals was compared with the quality of images reconstructed with data acquired at non-uniform intervals. Using the proposed method, even if only 70% of the regularly acquired radar sensor data was used, a SAR image having a correlation of 0.83 could be reconstructed.

## 1. Introduction

In recent years, studies on autonomous driving of vehicles or autonomous flight of unmanned aerial vehicles have been actively conducted. To realize the autonomous driving and flight technologies, the use of various sensors such as cameras, lidars, and radars is essential. Among these sensors, the radar sensor has the advantage of being robust to environmental changes [1]. In addition, as the radar sensor uses a higher frequency band and a wider bandwidth, the range resolution is greatly improved [2] and the size of the sensor is also reduced. With these advantages, radar sensors are considered essential sensors for autonomous driving and flight.

The main purpose of using the radar sensor is to determine the location information of an object. In addition to measuring the distance to the object, radar sensors have been used for various purposes. From radar sensor data, the type of the detected object can be identified [3], and even biometric information such as a person’s respiration rate or heart rate can be extracted [4]. Moreover, one of the most solicited functions of the radar sensor is its ability to image the surrounding environment. The most representative radar imaging technology is the synthetic aperture radar (SAR) image reconstruction method, which is widely used in aircrafts [5]. In airborne radar systems, algorithms such as the range-Doppler algorithm [6], chirp scaling algorithm [7], and range-migration algorithm (RMA) [8] have been used to reconstruct SAR images. To obtain high image quality from these SAR image reconstruction algorithms, the speed of the radar-equipped platform should be constant and the radar sensor data should be spatially uniformly acquired.

Recently, these SAR imaging technologies have been applied to small platforms such as automobiles [9] or drones [10,11]. For example, methods for reconstructing SAR images of cars parked in a parking lot were proposed in [12,13,14]. However, compared to aircrafts, it is difficult to meet the conditions of constant speed and regular data acquisition in small SAR platforms. Therefore, in this study, we propose a compressive sensing (CS)-based method for reconstructing the SAR image even if the radar sensor data are not acquired at regular spatial intervals. The feasibility of applying the CS technique to SAR image reconstruction has been demonstrated in several studies [15,16]. For example, the authors in [17] used the CS-based technique to determine the presence of targets in the same line of sight. In addition, methods of improving accuracy and resolution in angle estimation using CS-based algorithms were also introduced in [18,19]. Recently, in [20,21], CS-based high-resolution SAR image reconstruction methods in the frequency-modulated continuous wave (FMCW) radar system were presented. Unlike the mentioned studies, we use a CS-based signal recovery method to generate sensor data from irregularly acquired data.

In this study, we used the RMA as a basic SAR image reconstruction method, which is known to be effective in generating near-field SAR images [22]. The potential for improving the performance of RMA by applying CS-based signal recovery to signals received from sparsely arranged antenna elements was reported in [23]. In our work, we solved the non-uniform data acquisition problem by using the l1-norm minimization [24]. Based on the fact that the time-domain radar sensor data are sparse, the processes of Fourier transform and inverse Fourier transform of the conventional RMA algorithm were replaced by the CS-based signal recovery method. In addition, by applying the proposed signal recovery method to each of the in-phase and quadrature (I/Q) components of the irregularly acquired radar sensor data, we were able to reduce the phase distortion of the restored signal. To evaluate the performance of the proposed method, experiments were conducted with a millimeter-wave band FMCW radar sensor. Then, the quality of the SAR image reconstructed using the entire data was compared with the quality of the SAR images reconstructed from randomly acquired data.

The remainder of this paper is organized as follows. We introduce the basic principles of the FMCW radar system and explain the fundamentals of the conventional SAR image reconstruction method in Section 2. In Section 3, we propose a method for applying the CS-based signal recovery algorithm to the SAR image reconstruction. Then, the performance of the proposed method is verified through actual experimental results in Section 4. Finally, we conclude this paper in Section 5.

## 2. SAR Image Reconstruction with FMCW Radar Sensor Data

### 2.1. Principles of FMCW Radar System

**Figure 1:**
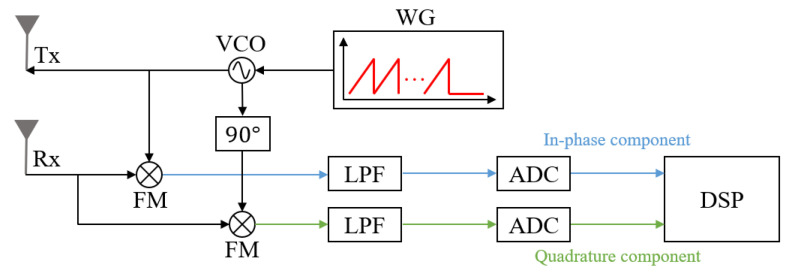
Block diagram of the FMCW radar system.

In general, the FMCW radar system consists of a waveform generator (WG), voltage-controlled oscillator (VCO), transmitting antenna (Tx), receiving antenna (Rx), frequency mixer (FM), low-pass filter (LPF), analog-to-digital converter (ADC), and digital signal processor (DSP), as shown in Figure 1. As shown in the figure, the waveform generator produces a series of chirps whose frequency increases linearly with time. In other words, a total of $N_{C}$ chirps are generated every cycle $T_{P}$ in the waveform generator. This waveform is frequency-modulated with the center frequency $f_{c}$ and then radiated through the transmitting antenna. The *n*-th ($n=1,\;2,\;\ldots ,\;N_{C}$) chirp in the transmitted waveform can be expressed as follows:

(1) $$\begin{array}{c} s_{n}\left(t\right)=\alpha_{n}\exp\left(j\left(2\pi \left(f_{c}-\frac{2n-1}{2}\Delta f\right)t+\pi \frac{\Delta f}{\Delta t}t^{2}+\phi_{n}\right)\right)\;\left(n-1\right)\Delta t<t<n\Delta t, \end{array}$$

where $\alpha_{n}$ and $\phi_{n}$ represent the amplitude and phase offset of the *n*-th chirp. In addition, Δt and Δf denote the chirp duration and bandwidth of each chirp.

The transmitted waveform in (1) is reflected from various objects in the antenna’s field of view. The received waveform can be expressed as follows:

(2) $$\begin{array}{c} r_{n}\left(t\right)=\sum_{k=1}^{K}\beta_{n,\;k}\exp\left(j\left(2\pi \left(f_{c}+f_{d,\;k}-\frac{2n-1}{2}\Delta f\right)\left(t-t_{d,\;k}\right)\right.\right.\\ \left.\left.+\pi \frac{\Delta f}{\Delta t}{\left(t-t_{d,\;k}\right)}^{2}+\phi_{n}\right)\right), \end{array}$$

where $\beta_{n,\;k}$ represents the amplitude of the received signal reflected from the *k*-th(k=1,2,…,K) object. In addition, $t_{d,\;k}$ denotes the time delay caused by the distance between the radar and the *k*-th object, and $f_{d,\;k}$ denotes the Doppler shift generated by the velocity of the *k*-th object.

Then, $r_{n}\left(t\right)$ is multiplied by $s_{n}\left(t\right)$ in the frequency mixer, and the output of the frequency mixer is passed through the low-pass filter, as shown in Figure 1. The output of the low-pass filter can be expressed as follows:

(3) $$\begin{array}{ccc} m_{n}\left(t\right) & = & {\left\{s_{n}\left(t\right)r_{n}\left(t\right)\right\}}_{\mathrm{LPF}}\\ & ≅ & \frac{1}{2}\sum_{k=1}^{K}\alpha_{n}\beta_{n,\;k}\exp\left(j\left(2\pi \left(t_{d,\;k}\frac{\Delta f}{\Delta t}-f_{d,\;k}\right)t+\psi_{n}\right)\right), \end{array}$$

where $\psi_{n}$ is the phase offset of the filtered signal. This baseband signal is composed of the sum of cosine waves, and the frequency of each cosine wave contains the distance and velocity information of each object [25].

The output of the low-pass filter is sampled while passing through the analog-to-digital converter, and the time-sampled signal in each chirp can be expressed as follows:

(4) $$\begin{array}{ccc} m_{n} & = & \left[m_{n}\left(t=T_{S}\right),\;m_{n}\left(t=2T_{S}\right),\ldots ,\;m_{n}\left(T=N_{S}T_{S}\right)\right]\\ & = & \left[m_{n}\left[1\right],\;m_{n}\left[2\right],\ldots ,\;m_{n}\left[N_{S}\right]\right], \end{array}$$

where $T_{S}$ is the sampling period and $N_{S}$ is the total number of time samples. In general, using all $N_{C}$ chirps increases the signal-to-noise ratio (SNR) for the object, but also increases the time it takes to acquire and store sensor data. Therefore, in this study, only the sampled data from the first chirp are used to reduce the time required to generate the SAR image. The effect of increasing the SNR is achieved by generating an image considering all time samples from each chirp, which is explained in Section 2.2.

### 2.2. Fundamentals of Range Migration Algorithm

**Figure 2:**
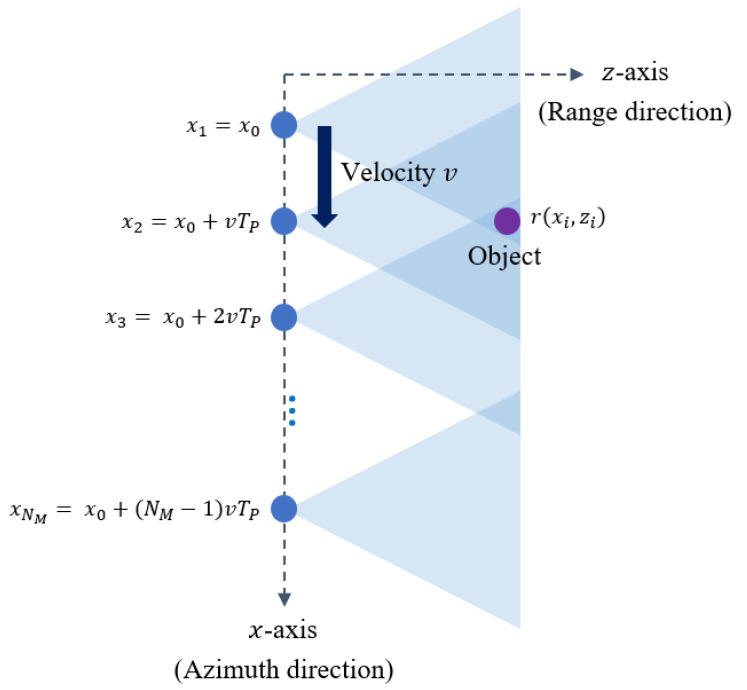
Movement of the SAR platform in the azimuth direction.

In this study, we use the RMA-based method to reconstruct the SAR image. The RMA method is efficient for near-field SAR image generation [22], which is suitable for SAR image reconstruction on small radar-equipped platforms. Reconstructing the SAR image in the RMA method is equivalent to calculating the reflectivity at each point $\left(x_{i},\;z_{i}\right)$. The reflectivity depends on the radar cross section of the object and the distance between the radar and the object, which greatly affects the quality of the SAR image reconstruction result. First, we assume that the initial position of the SAR platform is $x_{0}$ and that it moves along the *x*-axis with a constant velocity *v*, which is shown in Figure 2. In the figure, the position of the SAR platform can be expressed as $x_{i}=x_{0}+\left(i-1\right)vT_{P}\;$$\left(i=1,\;2,\;\ldots ,\;N_{M}\right)$, where $N_{M}$ denotes the total number of measurements.

In general, to reconstruct the results of radar detection as an image, data acquisition is required at regular spatial intervals along a straight line. In other words, the signal of (4) is obtained at each point $x_{i}$ for every $T_{P}$. If the first chirp signal received at *i*-th point is expressed as $m^{\left(i\right)}$, the initial input of the SAR image reconstruction can be expressed as follows:

(5) $$\begin{array}{c} M={\left[m^{\left(1\right)},\;m^{\left(2\right)},\;\ldots ,\;m^{\left(N_{M}\right)}\right]}^{T}, \end{array}$$

where the size of M becomes $N_{M}\times N_{S}$. Then, we apply the Fast Fourier transform (FFT) in the *x*-axis direction on M, which can be expressed as F{M}. If we set the number of FFT points to be equal to $N_{M}$, the size of F{M} also becomes $N_{M}\times N_{S}$.

The next step in SAR image generation is the compensation of the amplitude and phase of F{M}. To compensate the amplitude, the FFT result must be multiplied by the wavenumber in the *z*-axis direction [26], which can be calculated as follows:

(6) $$\begin{array}{c} {k_{z}}^{2}={\left(2k\right)}^{2}-{k_{x}}^{2}, \end{array}$$

where *k* is the wavenumber, and $k_{x}$ is the wavenumber in the *x*-axis direction. Because the frequency of the FMCW radar signal changes with time, $k=\frac{2\pi f}{c}$ has a value that varies according to the effective bandwidth ${\Delta f}_{eff}$. Based on (6), the amplitude compensation matrix $K_{z}$ is generated, which has the size of $N_{M}\times N_{S}$. In addition, the phase for position $z_{i}$ in the *z*-axis direction can be compensated with the factor $e^{-jK_{z}z_{i}}$, which also has the size of $N_{M}\times N_{S}$. Therefore, the compensation for the amplitude and phase can be summarized as $F\left\{M\right\}⊗K_{z}⊗e^{-jK_{z}z_{i}}$, where ⊗ denotes the element-wise multiplication operator for matrices.

Finally, an inverse Fourier transform is applied to reconstruct the SAR image, which can be expressed as follows:

(7) $$\begin{array}{c} \hat{M}=F^{-1}\left\{F\left\{M\right\}⊗K_{z}⊗e^{-jK_{z}z_{i}}\right\}, \end{array}$$

where the size of $\hat{M}$ is equal to M. As mentioned in Section 2.1, to increase the SNR of the reconstructed SAR image at point $\left(x_{i},\;z_{i}\right)$, the process of adding reflectivities for all $N_{S}$ time samples is applied. Finally, the reflectivity at point $\left(x_{i},\;z_{i}\right)$ can be expressed as follows:

(8) $$\begin{array}{c} r\left(x_{i},\;z_{i}\right)=\sum_{q=1}^{N_{S}}\hat{M}\left(p=i,\;q\right), \end{array}$$

where $\hat{M}\left(p,\;q\right)$ denotes the (p,q)-th element of $\hat{M}$.

## 3. Proposed SAR Image Reconstruction with Compressive Sensing

**Figure 3:**
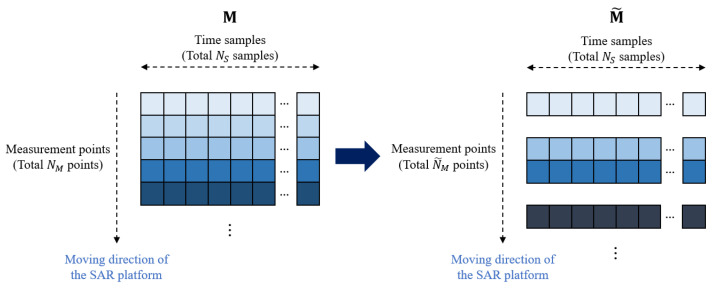
Regular and irregular data acquisition.

When the radar sensor data are received at all $N_{M}$ measurement points, the sensor data can be expressed as M in (5). However, if the SAR platform is not moving at a constant velocity, the distance between the two measurement points changes with time, as shown in Figure 3. When the regular data acquisition is not performed, the quality of the reconstructed SAR image deteriorates. Therefore, we propose a CS-based signal recovery method for restoring the image quality when the moving speed of the platform is not constant.

If we express the data matrix with irregular data acquisition as $\tilde{M}$, the matrix can be thought of as a matrix in which some values in M are filled with zeros. Therefore, the problem of restoring M from $\tilde{M}$ can be defined as the l1-norm minimization problem [24]. As shown in Figure 3, we assume that the size of $\tilde{M}$ is ${\tilde{N}}_{M}\times N_{S}$, where ${\tilde{N}}_{M}$ is less than $N_{M}$. If the received signal vector at all measurement points for the *j*-th time samples is expressed as $\tilde{M}\left(u,\;v=j\right)$, the signal vector $s^{*}$ we want to restore can be obtained by solving the minimization problem, which is expressed as follows:

(9) $$\begin{array}{ccc} s^{*} & = & \mathrm{argmin}\;{∥s∥}_{1}\\ \mathrm{subject}\;\mathrm{to}\;Fs & = & \tilde{M}\left(u,\;v=j\right), \end{array}$$

where ${∥\cdot ∥}_{1}$ denotes the l1-norm operator. In addition, the matrix F represents the inverse Fourier transform matrix of size ${\tilde{N}}_{M}\times N_{M}$. The problem in (9) can be re-expressed as a linear program problem, and it can be solved with the primal-dual interior point method [27]. Finally, by multiplying $s^{*}$ by the inverse Fourier transform matrix G of size $N_{M}\times N_{M}$ again, signals recovered in the time domain can be obtained, which are expressed as $Gs^{*}$.

If this process is repeated for all $N_{S}$ time samples, a signal matrix reconstructed in the time domain ${\tilde{M}}^{*}$ is obtained. Then, the SAR image can be reconstructed by applying the same amplitude and phase compensation method as in (7) to ${\tilde{M}}^{*}$, which can be expressed as follows:

(10) $$\begin{array}{c} {\hat{M}}^{*}={\tilde{M}}^{*}⊗K_{z}⊗e^{-jK_{z}z_{i}}. \end{array}$$

Finally, the reflectivity at point $\left(x_{i},\;z_{i}\right)$ can be expressed as:

(11) $$\begin{array}{c} r^{*}\left(x_{i},\;z_{i}\right)=\sum_{q=1}^{N_{S}}{\hat{M}}^{*}\left(p=i,\;q\right), \end{array}$$

where ${\hat{M}}^{*}\left(p,\;q\right)$ denotes the (p,q)-th element of ${\hat{M}}^{*}$. In (11), we also add reflectivities for all time samples to increase the SNR.

**Figure 4:**
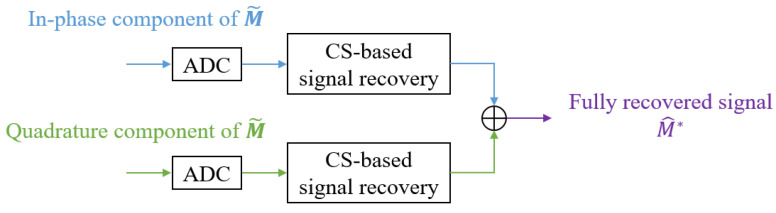
Proposed CS-based signal recovery method applied to each of the I/Q components.

Moreover, when the CS-based signal recovery is applied to a signal in which I/Q components are combined, phase information is not properly restored and the SAR image quality can be degraded. Thus, we apply the CS-based signal recovery method to each of the I/Q components in order to improve image quality, as shown in Figure 4.

**Figure 5:**
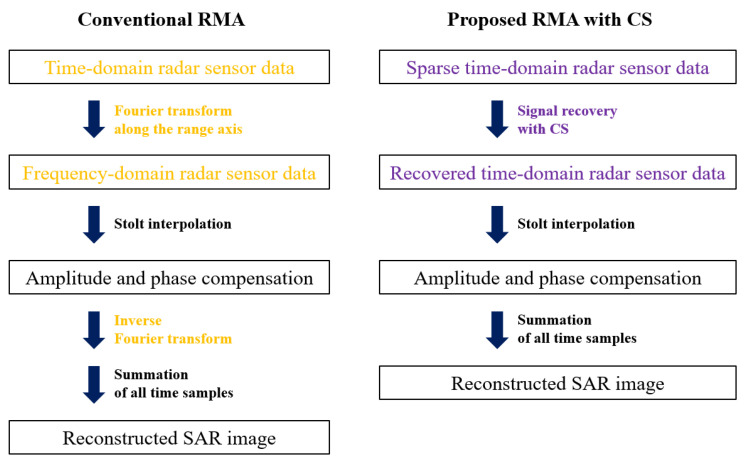
Conventional SAR image reconstruction using the RMA and the proposed CS-based SAR image reconstruction method.

In Figure 5, the processes of the conventional SAR image reconstruction using the RMA and the proposed SAR image reconstruction using the CS-based algorithm are summarized. As shown in the figure, the Fourier transform and inverse Fourier transform are sequentially applied in the conventional SAR image reconstruction. However, those processes were replaced by the CS-based signal restoration in our proposed method.

## 4. Performance Evaluation

### 4.1. Radar Sensor Data Acquisition

**Figure 6:**
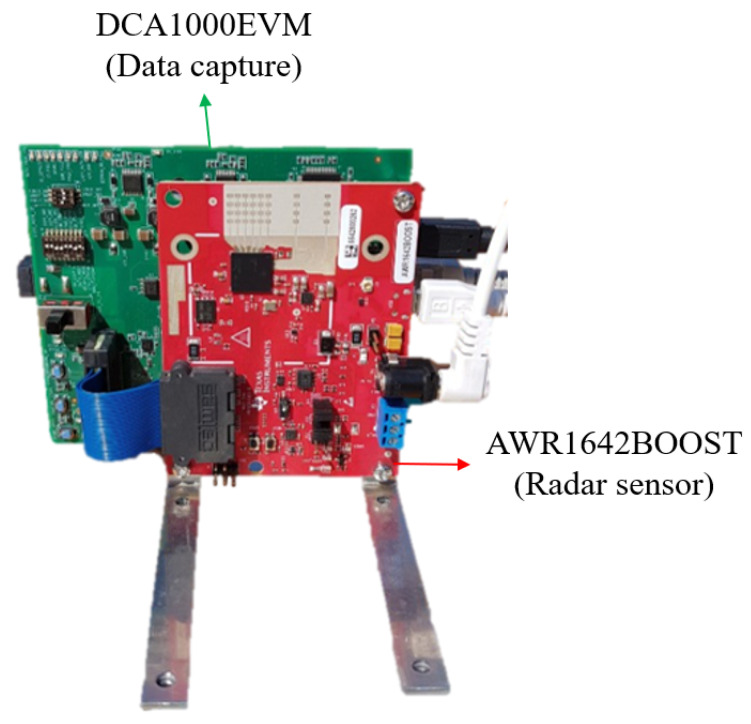
Radar sensor module used in our experiments.

In this section, we evaluate performance by applying the proposed method to actual radar sensor data. In our experiments, we used the FMCW radar sensor module developed by Texas Instruments. It consists of a 76–81 GHz automotive radar sensor (i.e., AWR1642BOOST) and a real-time data-capture adapter (i.e., DCA1000EVM), as shown in Figure 6. In addition, the radar parameter values used in our experiments are summarized in Table 1.

**Figure 7:**
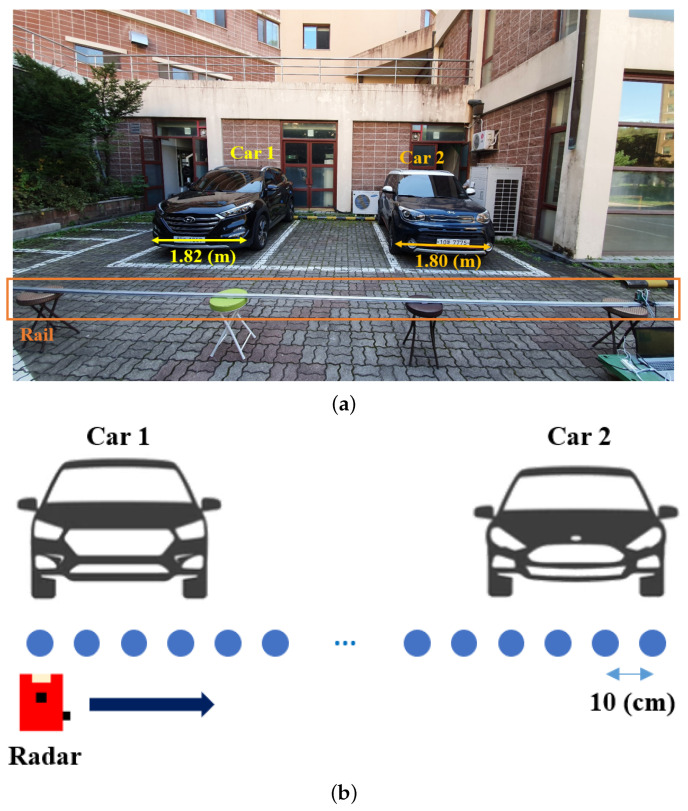
(**a**) Experimental environment; (**b**) radar sensor data acquired every 10 cm.

Using the radar sensor with these specifications, we conducted radar signal measurements in an outdoor parking lot, which is shown in Figure 7a. In the experimental environment, two different cars (i.e., Tucson made by Hyundai Motor Company and Soul made by Kia Motors) were parked side by side, and the radar sensor was mounted on a rail. The rail was placed parallel to the front bumpers of both cars at a distance of 2 m. The radar sensor was installed on the rail so that the boresight direction of the antenna system was directed toward the car. In other words, the moving direction of the radar and the boresight direction of the antenna were perpendicular to each other. Then, the radar moved at a constant speed from left to right on the rail, and we acquired and stored sensor data at intervals of 10 cm using the data-capture adapter. We set the data acquisition interval to 10 cm, in consideration of the range resolution presented in Table 1. In summary, the radar sensor data were acquired every 10 cm using this measurement system, as shown in Figure 7b. Because the distance from the left side of Car 1 to the right side of Car 2 is 6.4 m, there were a total of 64 measurement points between them (i.e., $N_{M}=64$).

### 4.2. SAR Image Reconstruction Results

**Figure 8:**
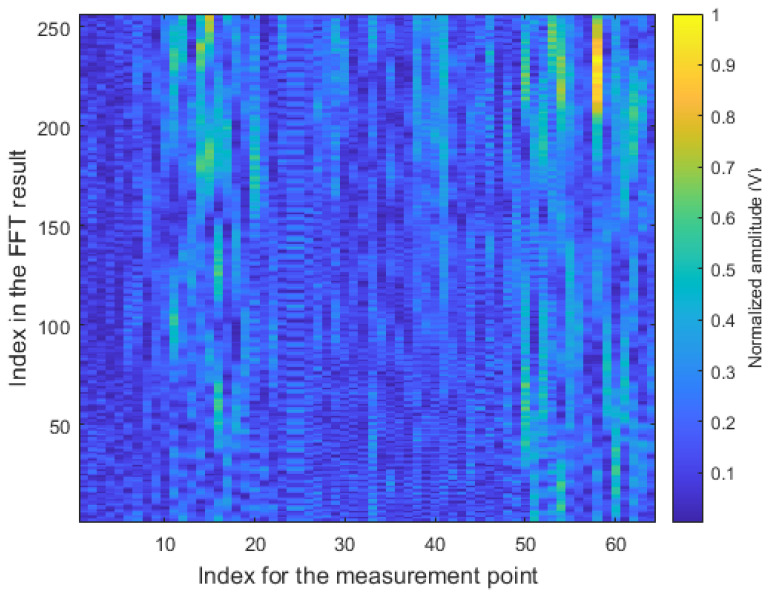
Fourier transform result of the radar sensor data acquired from all measurement points.

**Figure 9:**
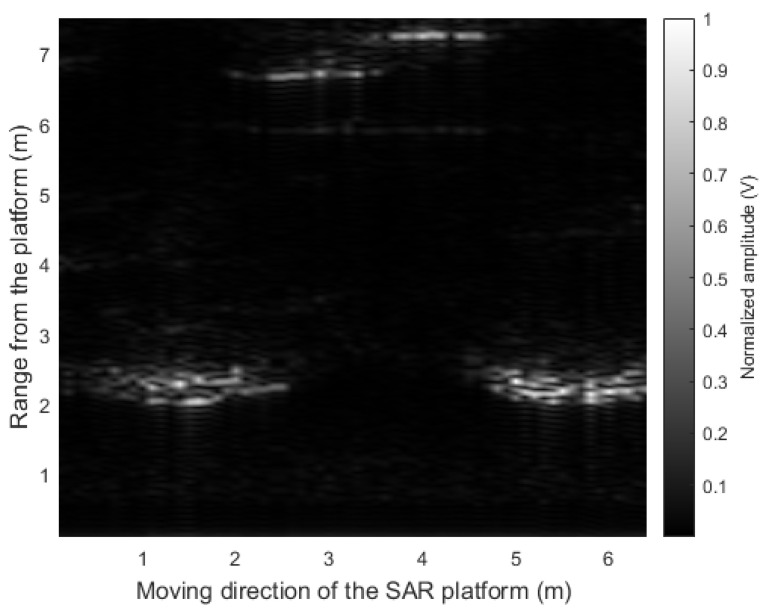
SAR image reconstruction result using the sensor data acquired from all measurement points (i.e., using the conventional RMA method).

First, we reconstructed the SAR image using all the acquired sensor data, which means that the conventional RMA was applied. We applied the FFT to the acquired radar sensor data in the *x*-axis direction, which is equivalent to F{M}. Figure 8 shows the Fourier transform result of the radar sensor data acquired from a total of 64 points. As shown in the figure, components with significant magnitudes exist sparsely in the FFT-processed matrix data. For this matrix data, the amplitude and phase compensation method described in Section 2.2 was applied, and then the inverse Fourier transform was applied again. When reconstructing the SAR images, the position in the range direction, $z_{i}$, was set from 0 to 5 m at 10 cm intervals. Finally, the SAR image reconstruction result using the conventional RMA is shown in Figure 9. As shown in the figure, the radar signals are strongly reflected on the front faces of the two cars, and this is clearly shown in the SAR image reconstruction results. In addition, the figure shows strong reflections of radar signals even from the wall between the cars.

**Figure 10:**
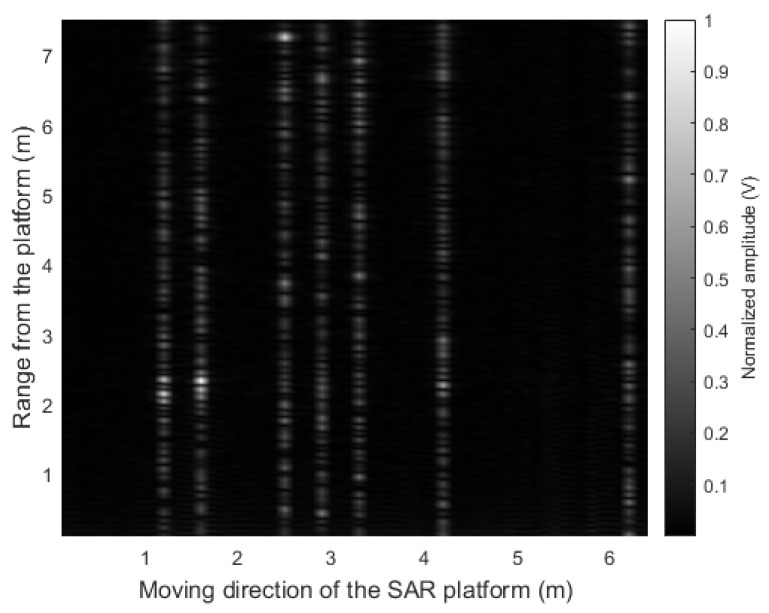
SAR image reconstruction result: when the CS-based signal recovery is applied to the sensor data in which the I/Q components are combined.

Next, instead of using the sensor data acquired from all measurement points, the SAR image was reconstructed using only data acquired from randomly selected measurement points. That is, it corresponds to the case where the radar sensor data were acquired when the speed of the SAR platform was not constant and varied. For example, we reconstructed the SAR image assuming that 10% of the total measurement data were lost. Then, the proposed CS-based signal recovery method in (9) was applied to the radar sensor data in which the I/Q components were combined; the following SAR reconstruction result is shown in Figure 10. The white lines in the figure indicate randomly selected and discarded measurement points, and correspond to the sensor data restored by the CS-based method. As shown in the figure, the SAR image was not properly reconstructed because the amplitude and phase components of the radar sensor data were not properly restored by the CS-based signal recovery.

**Figure 11:**
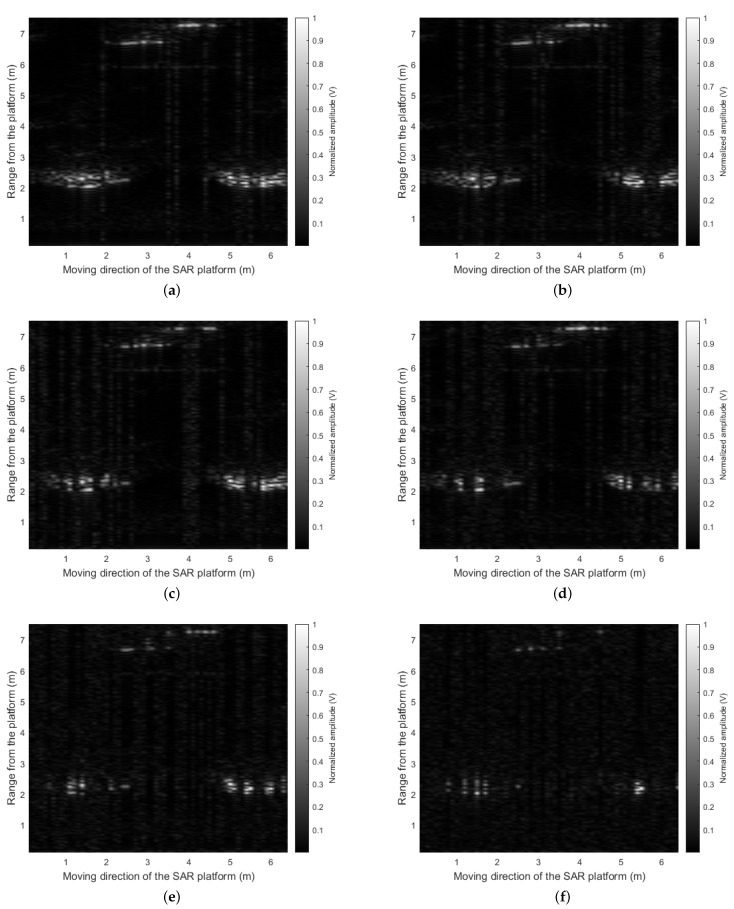
SAR image reconstruction result using the CS-based signal recovery: (**a**) 90% of the total radar sensor data used; (**b**) 80% of the total radar sensor data used; (**c**) 70% of the total radar sensor data used; (**d**) 60% of the total radar sensor data used; (**e**) 40% of the total radar sensor data used; (**f**) 20% of the total radar sensor data used.

Therefore, we finally applied the proposed CS-based signal recovery technique to each of the I/Q components, as shown in Figure 4. Figure 11 shows the SAR image reconstruction results when 90%, 80%, 70%, and 60% of the total radar sensor data were randomly selected and used for the signal recovery, respectively. When comparing Figure 10 and Figure 11a, the quality of the image reconstruction result was greatly improved as compared to when the proposed signal recovery was applied to the signal in which the I/Q components were combined. Moreover, although the amount of the sensor data was reduced and non-uniform interval data acquisition was applied, the SAR image reconstruction results were similar to those in Figure 9. Because the acquired radar signal has a sparse characteristic in the FFT domain, as shown in Figure 8, the CS-based signal recovery can be effectively applied.

To quantitatively evaluate the performance of the proposed CS-based SAR image reconstruction method, we calculated the Pearson correlation coefficient [28] between the reconstructed SAR images and the reference SAR image (i.e., Figure 9). In other words, the degree of similarity between $\rho (x_{i},\;z_{i})$ and $\rho^{*}\left(x_{i},\;z_{i}\right)$ for all points $\left(x_{i},\;z_{i}\right)$ is evaluated as the value of the correlation coefficient. Table 2 shows the calculated correlation coefficient values for each case. As given in the table, the correlation decreases as the amount of sensor data used decreases. However, even if 30% of regularly acquired data are lost, the SAR image reconstructed with the remaining data has a correlation of 0.83 with respect to the reference SAR image. Therefore, with our proposed method, even if the radar-equipped SAR platform does not acquire sensor data at spatially regular intervals, the SAR image can be effectively reconstructed.

## 5. Conclusions

In this paper, we proposed the CS-based signal recovery method for reconstructing SAR images from irregular sensor data acquisition. First, we applied the l1-norm minimization problem to restore the sensor data from non-uniformly acquired sensor data. In addition, by applying the proposed CS-based recovery method to each of the I/Q components of the radar sensor data, the effect of distortion occurring in the phase of the signal was also reduced. Finally, the performance of the proposed method was verified using the data obtained from the automotive FMCW radar sensor. The SAR image was reconstructed using only a part of the entire radar sensor data, and the similarity with the reference SAR image was calculated through the correlation coefficient. The proposed method is expected to be efficiently used in small SAR platforms (e.g., vehicles or drones) that hardly move at a constant speed.

## Figures and Tables

**Table 1:** Radar parameters used in the FMCW radar system.

Radar Parameters	Value
Center frequency, $f_{c}$	78.79 (GHz)
Bandwidth, Δf	3.57 (GHz)
Effective bandwidth, ${\Delta f}_{eff}$	1.79 (GHz)
Sweep time, Δt	151 (μs)
The number of time samples in each chirp, $N_{S}$	256
The number of chirps, $N_{C}$	8
Sampling frequency	10 (MHz)
Range resolution	8.4 (cm)
Velocity resolution	0.79 (m/s)

**Table 2:** Pearson correlation coefficient [28] according to the ratio of the radar sensor data used.

Ratio of Radar Sensor Data Used	Correlation Coefficient
90%	0.93
80%	0.89
70%	0.83
60%	0.76
50%	0.69
40%	0.66
30%	0.59
20%	0.38
10%	0.28

## Data Availability

Data sharing not applicable.

## Abbreviations

The following abbreviations are used in this manuscript:

ADC	Analog-to-digital converter
CS	Compressive sensing
DSP	Digital signal processor
FFT	Fast Fourier transform
FM	Frequency mixer
FMCW	Frequency-modulated continuous wave
I/Q	In-phase and quadrature
LPF	Low-pass filter
RMA	Range migration algorithm
Rx	Receiving antenna
SAR	Synthetic aperture radar
SNR	Signal-to-noise ratio
Tx	Transmitting antenna
VCO	Voltage-controlled oscillator
WG	Waveform generator
